# Intra-tidal PaO_2_ oscillations associated with mechanical ventilation: a pilot study to identify discrete morphologies in a porcine model

**DOI:** 10.1186/s40635-023-00544-0

**Published:** 2023-09-06

**Authors:** John N. Cronin, Douglas C. Crockett, Gaetano Perchiazzi, Andrew D. Farmery, Luigi Camporota, Federico Formenti

**Affiliations:** 1 Department of Anaesthesia and Perioperative Medicine, St. Thomas’ Hospital, Guy’s and St. Thomas’ NHS Foundation Trust, Westminster Bridge Road, London, SE1 7EH UK; 2 Faculty of Life Sciences and Medicine, King’s College London, London, UK; 3 Nuffield Department of Clinical Neurosciences, University of Oxford, Oxford, UK; 4 Hedenstierna Laboratory, Department of Surgical Sciences, University of Uppsala, Uppsala, Sweden; 5 Department of Intensive Care, Guy’s and St. Thomas’ NHS Foundation Trust, London, UK

**Keywords:** Acute respiratory distress syndrome, Mechanical ventilation, Arterial oxygen tension, Oxygen oscillations, Functional principal component analysis

## Abstract

**Background:**

Within-breath oscillations in arterial oxygen tension (PaO_2_) can be detected using fast responding intra-arterial oxygen sensors in animal models. These PaO_2_ signals, which rise in inspiration and fall in expiration, may represent cyclical recruitment/derecruitment and, therefore, a potential clinical monitor to allow titration of ventilator settings in lung injury. However, in hypovolaemia models, these oscillations have the potential to become inverted, such that they decline, rather than rise, in inspiration. This inversion suggests multiple aetiologies may underlie these oscillations. A correct interpretation of the various PaO_2_ oscillation morphologies is essential to translate this signal into a monitoring tool for clinical practice. We present a pilot study to demonstrate the feasibility of a new analysis method to identify these morphologies.

**Methods:**

Seven domestic pigs (average weight 31.1 kg) were studied under general anaesthesia with muscle relaxation and mechanical ventilation. Three underwent saline-lavage lung injury and four were uninjured. Variations in PEEP, tidal volume and presence/absence of lung injury were used to induce different morphologies of PaO_2_ oscillation. Functional principal component analysis and *k*-means clustering were employed to separate PaO_2_ oscillations into distinct morphologies, and the cardiorespiratory physiology associated with these PaO_2_ morphologies was compared.

**Results:**

PaO_2_ oscillations from 73 ventilatory conditions were included. Five functional principal components were sufficient to explain ≥ 95% of the variance of the recorded PaO_2_ signals. From these, five unique morphologies of PaO_2_ oscillation were identified, ranging from those which increased in inspiration and decreased in expiration, through to those which decreased in inspiration and increased in expiration. This progression was associated with the estimates of the first functional principal component (*P* < 0.001, *R*^2^ = 0.88). Intermediate morphologies demonstrated waveforms with two peaks and troughs per breath. The progression towards inverted oscillations was associated with increased pulse pressure variation (*P* = 0.03).

**Conclusions:**

Functional principal component analysis and *k*-means clustering are appropriate to identify unique morphologies of PaO_2_ waveform associated with distinct cardiorespiratory physiology. We demonstrated novel intermediate morphologies of PaO_2_ waveform, which may represent a development of zone 2 physiologies within the lung. Future studies of PaO_2_ oscillations and modelling should aim to understand the aetiologies of these morphologies.

**Supplementary Information:**

The online version contains supplementary material available at 10.1186/s40635-023-00544-0.

## Background

The acute respiratory distress syndrome (ARDS) is a condition with significant morbidity and mortality. Although mechanical ventilation is often necessary in patients with ARDS, it can be associated with progression of lung injury via multiple mechanisms, including cyclical recruitment/derecruitment (R/D) of diseased and unstable alveolar units [1].

One promising avenue of research to minimise cyclical R/D is via analysis of intra-tidal variation in arterial oxygen tension (PaO_2_) utilising fast responding intra-arterial fibre optic sensors. In rabbit and porcine models of ARDS, mechanical ventilation settings that predispose to cyclical R/D (for example, large tidal volumes and low respiratory rates) were associated with large increases followed by large decreases (oscillations) in PaO_2_ within the time period of a single tidal breath [2–7]. It was, therefore, hypothesised that cyclical R/D caused intra-tidal PaO_2_ oscillations via varying shunt fraction through the time course of a single mechanical ventilatory breath. However, this hypothesis is not yet conclusively supported by experimental evidence. For example, cyclical R/D, as quantified by dynamic computed tomography (CT), was not associated with the amplitudes of PaO_2_ oscillations in pigs either with [8] or without experimental lung injury [9].

The cyclical R/D hypothesis implies that, within a single breath, the ratio between the amount of time the PaO_2_ is above its mean value to that where it is below the mean value is related to the inspiratory:expiratory (I:E) ratio. In other words, if the I:E ratio is 1:2, the PaO_2_ would be expected to be “high” for one-third of the breath and “low” for two-thirds, equivalent to the amount of time the shunt fraction was low and high. In the presence of hypovolaemia, however, this ratio was inverted i. e. PaO_2_ was above the mean value for more than half of the breath despite I:E being fixed at 1:2. Such inverted oscillations were termed “perfusion-dependent” as opposed to the “ventilation-dependent” oscillations suggested by the cyclical R/D hypothesis [10].

Such findings are problematic for the proposed clinical use of fast responding PaO_2_ to titrate mechanical ventilation settings against cyclical R/D in lung injury. It is apparent that in the case of perfusion-dependent oscillations, the amplitude of the oscillation will not necessarily be solely related to cyclical R/D but other factors, including intra-tidal variation in regional ventilation, regional and global lung perfusion, and oxygen utilisation by the animal within the time course of a single breath.

Clinical application of these fast-responding PaO_2_ sensors is, therefore, dependent upon further understanding of the morphology and aetiologies of these PaO_2_ oscillations and their dependence on heart–lung interactions during positive pressure mechanical ventilation. To further identify these PaO_2_ morphologies, we undertook a pilot study to assess the feasibility of a novel PaO_2_ signal analysis approach in a porcine model. We used functional principal component analysis and *k*-means clustering to identify unique morphologies and attempted to correlate them with cardiopulmonary variables, with the aim to provide a theoretical foundation for future clinical studies on PaO_2_ oscillations.

## Methods

Seven domestic pigs (weight 31.1 SD 1.6 kg, 6 male, 1 female) were studied under general anaesthesia and muscle relaxation at the Hedenstierna Laboratory, University of Uppsala, Sweden. Ethical approval was granted by the Uppsala Regional Animal Research Ethics Committee (ref. C98/16). The relevant sections of the ARRIVE guidelines [11] were adhered to.

### Experimental protocol

Following premedication with intramuscular xylazine 2 mg/kg, ketamine 20 mg/kg and midazolam 0.5 mg/kg, an ear vein was cannulated, and general anaesthesia commenced and maintained with an infusion of ketamine 32 mg/kg/h, fentanyl 4 mcg/kg/h and midazolam 0.16 mg/kg/h. Muscle relaxation was maintained with an intravenous infusion of rocuronium 30–100 mg/h titrated against spontaneous respiratory effort. Adequacy of anaesthesia was confirmed by absence of reaction to painful stimulation between the front hooves, and absence of any signs of sympathetic stimulation after administration of rocuronium. Aliquots of fentanyl 100–300 mcg were administered as required.

Following induction of general anaesthesia, an endotracheal tube was placed via tracheostomy and mechanical ventilation commenced using a Servo-I ventilator (Maquet, Rastatt, Germany). Immediately following tracheostomy, to maintain normoxia and normocapnia, the ventilation settings were fixed to volume control mode, tidal volume 8 mL/kg, respiratory rate 25/min, PEEP 5 cmH_2_O and FiO_2_ sufficient to maintain peripheral oxygen saturations above 92%. Settings were subsequently adjusted for the recording of PaO_2_ oscillations (see below). Ringer’s lactate solution was infused at 20 mL/kg/h for the first hour then reduced to 10 mL/kg/h for the remainder of the experiment.

These experiments were performed on a subset of animals included in a previously reported study [12] in which the indwelling fast responding PaO_2_ sensor was available. In this previous study, animals were randomised to either the lung-injury or uninjured group using simple randomisation. In the lung-injury group, the saline-lavage surfactant depletion technique of Lachmann [13] was used aiming for a PaO_2_/FiO_2_ ratio of 20–26.7 kPa (150–200 mmHg). 30 mL/kg of warmed saline was administered intratracheally until the PaO_2_ fell to less than 6.6 kPa (50 mmHg) or mean pulmonary artery pressure rose above 40 mmHg, at which point the lavage fluid was drained from the animal’s lungs and mechanical ventilation was restarted. The lavage process was repeated if PaO_2_ subsequently rose above 26.7 kPa (200 mmHg). PaO_2_ was recorded prior to each lavage, immediately after restarting ventilation and subsequently at 30 s intervals. FiO_2_ was fixed at 1.0 for the entire lavage procedure. The currently reported experiments were performed after induction of lung injury (if applicable), but prior to transfer to the CT scanner, where the previously reported study [12] was performed. Aside from presence/absence of experimental lung injury, there was no section of the protocol for either set of experiments that would adversely affect the results of the other study.

Each animal was ventilated at 12 different ventilatory conditions, representing all combinations of PEEP 5, 8, 10 and 12 cmH_2_O and tidal volume 7, 10 and 15 mL/kg. For each animal the order of ventilatory conditions was randomised using simple randomisation on pre-printed case record forms. Given the exploratory nature of this study the researchers were not blinded to group (lung-injury vs uninjured) allocation or the order of ventilatory conditions. Each animal was ventilated at each condition with respiratory rate 10/min, inspiratory:expiratory ratio 1:2 in pressure control mode with FiO_2_ sufficient to maintain PaO_2_ between 13.3 and 66.5 kPa (100 to 500 mmHg). This lower limit was chosen to ensure haemoglobin was fully saturated for the duration of the experiments so any intra-tidal variation in arterial blood oxygen content was directly proportional to PaO_2_. If this constraint is not observed, then the PaO_2_ waveform is non-linearly damped, because any intra-tidal variation in arterial oxygen content would to some extent be buffered by O_2_ release/absorption by haemoglobin [14]. The upper PaO_2_ limit of 66.5 kPa (500 mmHg) represented the upper limit of the PaO_2_ sensor used.

This was a pilot study to investigate the technique used to identify different PaO_2_ oscillation morphologies. Therefore, sample size was chosen to be sufficient to generate multiple morphologies of PaO_2_ oscillation rather than to demonstrate statistically significant differences between any identified PaO_2_ morphology. Based upon the results of previous experiments [8, 9] using fast-acting intra-arterial sensors to detect PaO_2_ oscillations, between 5 and 8 pigs would be sufficient to demonstrate a wide variety of PaO_2_ oscillation morphologies.

### Measurements

The left common carotid artery was cannulated using a standard 20G 80 mm arterial cannula (LeaderCath, Vygon, Ecouen, France). Through this cannula, a fibreoptic PaO_2_ sensor with a response time of < 100 ms was introduced and interfaced to an OxyLite Pro (Oxford Optronix, Oxford, UK). These sensors have been described previously [15, 16]. The analogue output from these sensors was continuously recorded (PowerLab and LabChart, ADInstruments, Dunedin, New Zealand) at a sampling frequency of 10 Hz.

Systemic arterial blood pressure was measured using a catheter placed in a branch of the right external carotid artery. A central venous catheter was sited via the right internal jugular vein to measure central venous pressure. A pulmonary artery catheter (Criticath SP5107U, Merit Medical, South Jordan, UT, USA) was placed via the right internal jugular vein using standard flotation methods. All blood pressure waveforms, as well as ECG and tail pulse oximetry, were continuously recorded (IntelliVue M8004A, Philips Healthcare, Best, Netherlands). Cardiac output was measured using a standard thermodilution bolus technique.

Respiratory waveforms including pressure and flow (with tidal volume calculated from the latter) were recorded using a Capnomac Ultima (Datex-Ohmeda, Madison, WI). Mechanical power was calculated using the simplified method for pressure control ventilation [17].

### Alignment of PaO_2_ oscillations with the phases of ventilation

For each ventilatory condition, the oscillations were aligned with the phases of ventilation (inspiration and expiration) by studying the PaO_2_ increase following the resumption of ventilation after a 20 s breath hold at end-expiration. The period between the start of inspiration (as measured by the airway pressure trace) and the first increase in PaO_2_, after the steady decline during the breath hold, was measured (Additional file 1: Fig. S1). This period (offset) was then retrospectively applied to the time axis of the PaO_2_ signal during mechanical ventilation immediately before the breath hold.

### Criteria for including PaO_2_ oscillations in analysis

PaO_2_ signals from certain ventilatory conditions were excluded from further analyses based upon pre-defined criteria. Signals with trough values less than 13.3 kPa (100 mmHg) were excluded to avoid non-linear dampening of the signal by O_2_ buffering by haemoglobin as described above. Others were excluded due to the signal-to-noise ratio of the PaO_2_ oscillations being less than 20 dB (empirically chosen based on preliminary results as being the minimum required to adequately detect the respiratory rate from the signal in 90% of cases). Finally, those oscillations where it was impossible to align the PaO_2_ signal with the phases of ventilation were excluded.

### PaO_2_ oscillation analysis

The PaO_2_ oscillations recorded for each ventilatory condition were assigned into one of several arbitrary clusters based upon the morphology of an aggregate single tidal breath. PaO_2_ oscillations from ten complete breaths were extracted for each ventilatory condition. Any linear trend in the ten-breath signal was removed. The ten breaths were combined into a single breath using a median of the PaO_2_ of each individual timepoint within the breaths to remove changes in PaO_2_ occurring at other frequencies, for example, the heart rate. This single breath was then normalized by converting the raw amplitude values (in mmHg) into multiples of the standard deviation of that breath. This conversion allowed comparison of the shape of the oscillations between different ventilatory conditions, without these being impacted by the amplitude of the oscillation or absolute PaO_2_ value.

### Functional principal component analysis

Functional principal component analysis (FPCA) [18] was then used to identify common signals in these normalised breaths. FPCA is a method of dimensionality reduction for complex time-series data, where the mean input PaO_2_ waveform is calculated and subtracted from each input waveform. A series of *eigenfunctions* is then created, such that all input waveforms can be reliably described by a weighted sum of these eigenfunctions. The mean waveform and eigenfunctions are constant between all input PaO_2_ waveforms, but the weights, termed principal component estimates, vary. Thus, a complex input waveform with 600 data points can be described by only a handful of principal components. The FPCA algorithm attempts to choose the optimum eigenfunctions, such that the maximum variance in the input waveforms can be explained with the fewest principal components. The *fdapace* [19] package in the R statistical software version 4.1.2 (R Foundation for Statistical Computing, Vienna, Austria) was used to calculate functional principal components.

### *k*-means clustering

The principal component estimates for each ventilatory condition were used to assign each ventilatory condition to a cluster using *k*-means clustering [20]. This attempts to partition each set of inputs (here the principal components) into unique clusters, thus each PaO_2_ input waveform is assigned to a single cluster. In our data, each point is multi-dimensional comprising a certain number of principal components. *k*-means clustering generates a certain number of *centre points,* such that, for each cluster, the sum of the squared distances between each set of input principal component estimates and its cluster centre is minimized. We used the iterative *k*-means clustering algorithm of Hartigan and Wong [21] to assign individual PaO_2_ waveforms to clusters.

Choice of both the number of input dimensions (number of principal components) as well as the number of clusters is problematic in *k*-means clustering as both can significantly affect the quality of the output. We initially set both the number of principal components and number of clusters as the number of principal components required to explain ≥ 95% of the variance in the input waveforms (semantically equivalent to a *R*^2^ value of ≥ 0.95 in other statistical models) and then optimised these based upon the Akaike Information Criterion (AIC) [22]. AIC was calculated based upon *k*-means clustering outputs [23]. Given that *k*-means clustering is stochastic, Monte Carlo simulation was performed over 10,000 iterations to generate AIC estimates for all potential cluster numbers ranging from zero to the maximum number of principal components identified.

### Statistical analyses

Differences in mean PaO_2_, PaO_2_ oscillation peak-to-trough height, presence of lung injury and cardiorespiratory variables between the identified PaO_2_ clusters were analysed using one-way analysis of variance or chi-squared testing as appropriate. Post-hoc testing was not performed between the individual clusters as this was a pilot study and inadequately powered to identify any such differences. Following visual allocation of cluster morphologies into a potential progression, we undertook linear regression analysis of the effects of this potential progression upon principal component estimates. In addition, an exploratory analysis of the effect of any unmeasured intra-animal effect upon cluster membership was performed. A mixed-effects linear model was developed with potential cluster progression as a continuous dependent variable, animal as a random-effects independent variable and other measured baseline cardiorespiratory parameters as fixed-effects dependent variables. The selection of fixed-effects variables was chosen, such that AIC was optimised. For these data, AIC was optimised when PaO_2_/FiO_2_ ratio and pulse pressure variation were included as fixed effects variables. Analyses were performed using the R statistical software version 4.1.2 (R Foundation for Statistical Computing, Vienna, Austria).

## Results

All seven animals completed the experiment, four of which were in the uninjured group and three in the lung-injury group. The number of lung lavages required to induce significant injury varied between the three lung-injured animals (1, 5 and 11 lavages) and the PaO_2_ trajectories for each lavage are provided in Additional file 2: Fig. S2.

Out of a total of 84 possible ventilatory conditions, 3 were excluded due to the trough of the PaO_2_ oscillation being below 13.3 kPa (100 mmHg), 3 were excluded due to the signal-to-noise ratio of the PaO_2_ oscillations being less than 20 dB, and 5 were excluded due to alignment of the PaO_2_ signal with the phases of ventilation not being possible (the PaO_2_ signal did not recover rapidly enough after the reinstitution of ventilation to allow a distinct change-point to be identified). Following exclusions, 73 ventilatory conditions (87% of total) were available for analysis.

The expiratory breath holds lasted 19.2 s (SD 1.6). Time delay between the restart of ventilation (as measured from the airway pressure trace) and the resultant change in the PaO_2_ signal was 1.23 s (0.23).

**Fig. 1:**
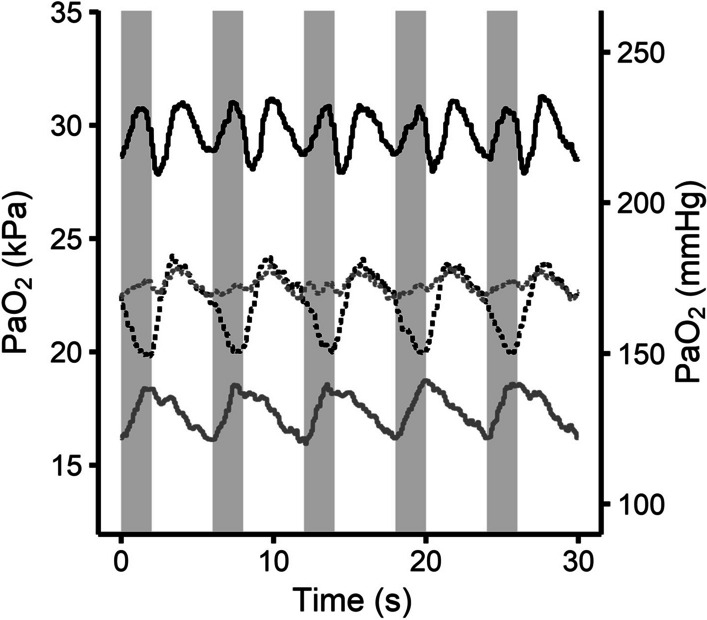
Example PaO_2_ waveforms aligned with the phases of ventilation. Waveforms taken from animals ventilated with either low PEEP (5 cmH_2_O) and high tidal volume (15 mL/kg) (black lines) or those ventilated with high PEEP (12 cmH_2_O) and low tidal volume (7 mL/kg) (grey lines). Solid lines denote animals without experimental lung injury and dashed lines those with. Light grey background denotes inspiration

**Fig. 2:**
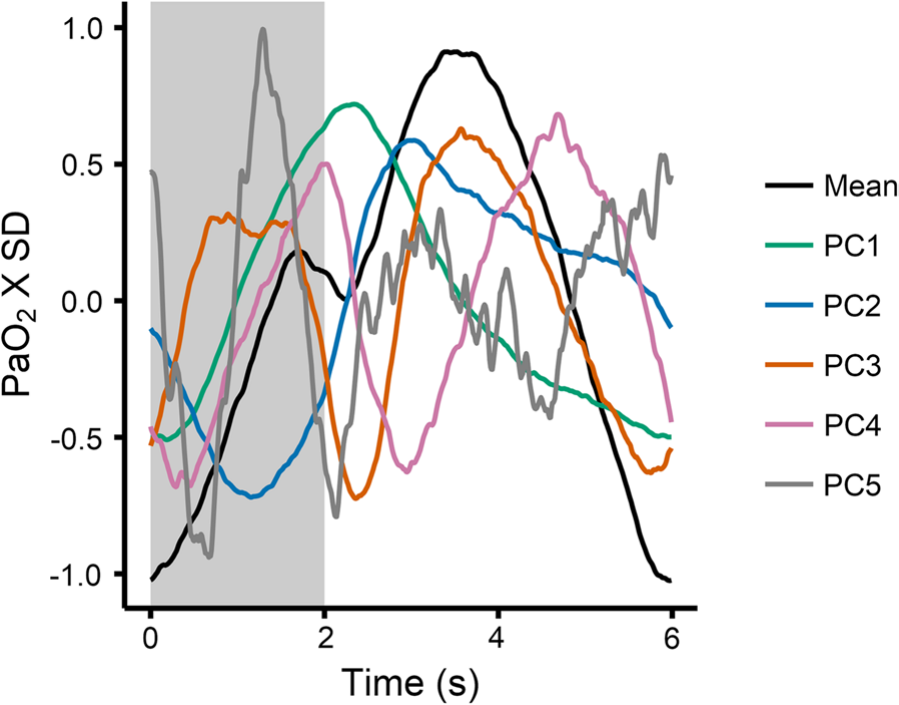
Mean value of all normalized PaO_2_ waveforms along with the first five eigenfunctions from functional principal component analysis (PC1 to PC5). Each individual normalized PaO_2_ waveform can be estimated as a sum of the mean waveform with varying multiples (principal component estimates) of each eigenfunction. Light grey background denotes inspiration

**Fig. 3:**
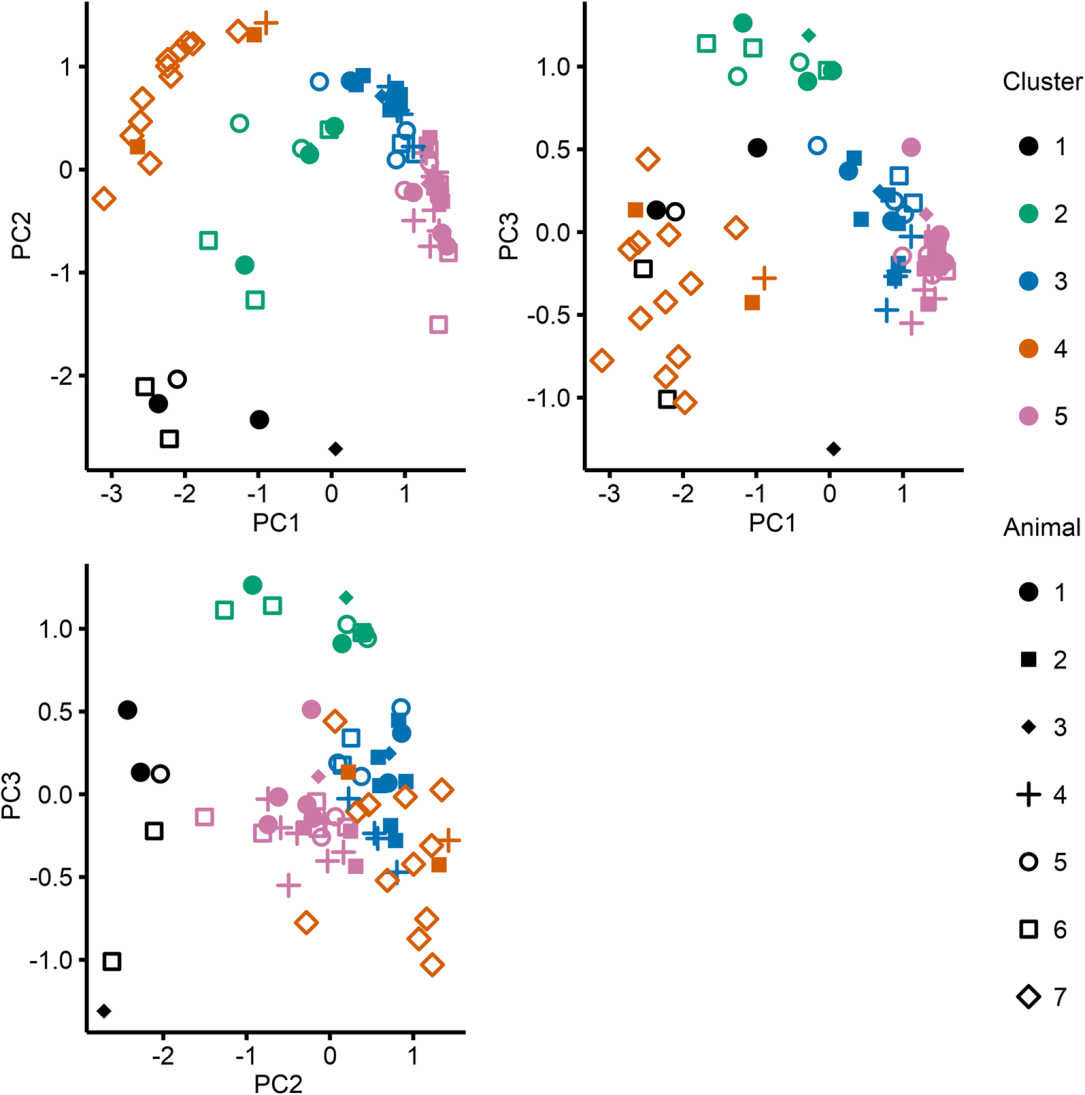
Estimates of the first three principal components (PC1 to PC3) for each individual ventilatory condition. A multi-dimensional *k*-means clustering algorithm was applied to these estimates to identify the PaO_2_ waveform cluster. The resulting clusters are denoted by colours and individual animals identified with shapes. An interactive 3D version of this figure is provided in Additional file 5: Fig. S5

**Fig. 4:**
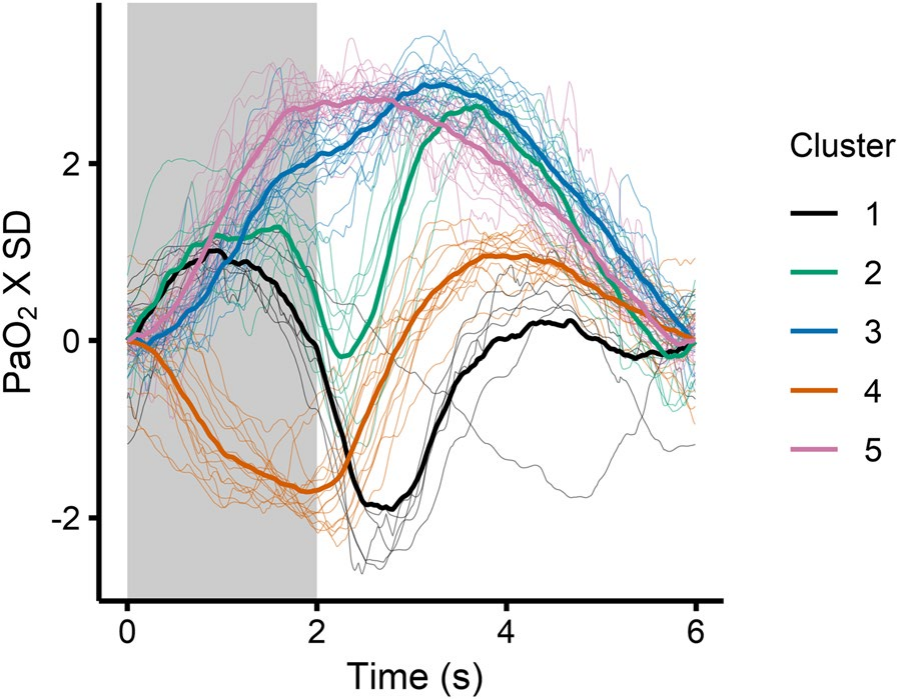
Allocation of PaO_2_ oscillations to each of the five identified clusters. Thin lines show all individual waveforms colour-coded by PaO_2_ oscillation cluster membership. Thick lines denote per-cluster mean, and light grey background inspiration

Discernible PaO_2_ oscillations were present in all ventilatory conditions studied, with some ventilatory conditions demonstrating PaO_2_ oscillations with two peaks and two troughs per breath (Fig. 1). Five functional principal components were required to explain 95% of the variance in the results (Additional file 3: Fig. S3). AIC was minimised with between 3, 4 and 5 clusters (Additional file 4: Fig. S4), and therefore, five clusters of PaO_2_ oscillations were identified based upon five principal components. Individual functional principal component waveforms are provided in Fig. 2 with the first three principal component estimates for each ventilatory condition provided in Fig. 3 and Additional file 5: Fig. S5. The allocation of PaO_2_ oscillations from each individual ventilatory condition to their relevant clusters is provided in Fig. 4.

A progression in PaO_2_ oscillation morphology was observed between the clusters ranging from cluster 5 which demonstrated non-inverted oscillations with the upslope during inspiration and the downslope during expiration through to cluster 4 where the downslope was during inspiration and the upslope during expiration. Intermediate clusters (3, 2 and 1 in order) demonstrated an upslope in early inspiration with a progressively large downslope in late inspiration followed by a second peak in expiration. This progression was also seen in the first principal component, which explained 56.9% of the total variance in the PaO_2_ waveforms (Additional file 6: Fig. S6, *P* < 0.001, *R*^2^ = 0.88).

Each individual cluster contained ventilatory conditions from at least three different animals, and all clusters contained ventilatory conditions from both injured and uninjured animals. Cluster 4 was predominantly composed of ventilatory conditions from a single animal (Additional file 7: Table S1).

Table 1 presents the haemodynamic parameters associated with each cluster. Given that cluster 4 was predominantly composed of data from a single injured animal, we also undertook a sensitivity analysis of the haemodynamic parameters with the exclusion of cluster 4 (Additional file 8: Table S2). In this sensitivity analysis, the variation in lung-injury indices and mean arterial pressure was no longer statistically significant amongst the clusters, however, but the variations in spirometry, cardiac output and pulse pressure variation persisted.

**Table 1:** Association between cluster membership and cardiorespiratory parameters

	Cluster 5	Cluster 3	Cluster 2	Cluster 1	Cluster 4	*P*
	Non-inverted ventilation-dependent		Intermediate		Inverted perfusion-dependent	
Lung injury						
Lung injured/uninjured ratio (% injured)	8/17 (32%)	5/13 (28%)	5/4 (56%)	3/3 (50%)	12/3 (80%)	0.02
PaO_2_/FiO_2_ ratio (kPa)	44 (11)	43 (10)	39 (9)	39 (9)	25 (11)	< 0.001
PaO_2_/FiO_2_ ratio (mmHg)	332 (84)	323 (74)	291 (66)	294 (70)	188 (83)	< 0.001
Lung mechanics						
Tidal volume (mL)	270 (114)	333 (88)	396 (117)	397 (134)	323 (107)	0.02
PEEP (cmH_2_O)	9 (3)	8 (2)	9 (3)	11 (1)	9 (3)	0.08
Plateau pressure (cmH_2_O)	19 (5)	20 (5)	26 (6)	30 (3)	22 (5)	< 0.001
Driving pressure (cmH_2_O)	10 (4)	12 (5)	17 (6)	18 (3)	12 (4)	< 0.001
Dynamic compliance (mL/cmH_2_O)	28 (8)	28 (8)	24 (9)	22 (8)	26 (5)	0.55
Mechanical power (J/min)	5.3 (3.8)	6.5 (2.6)	9.5 (3.3)	12.4 (3.1)	7.2 (3.9)	< 0.001
Haemodynamic variables						
Cardiac output (L/min)	4.74 (1.13)	4.20 (1.21)	5.36 (0.93)	5.39 (0.95)	4.88 (0.88)	0.05
Heart rate (/min)	125 (27)	110 (17)	146 (26)	125 (7)	132 (27)	0.01
Mean arterial pressure (mmHg)	102 (19)	98 (18)	100 (12)	92 (25)	80 (10)	0.004
Pulse pressure variation (%)	12.3 (5.7)	12.6 (5.3)	12.7 (4.0)	20.8 (4.2)	20.0 (8.3)	< 0.001
Central venous pressure (mmHg)	15 (2)	14 (2)	14 (1)	13 (2)	12 (2)	0.002
Pulmonary artery pressure (mmHg)	27 (5)	26 (5)	25 (4)	24 (5)	29 (5)	0.24
Systemic vascular resistance (dynes.s/cm^5^)	1546 (399)	1724 (430)	1348 (356)	1246 (556)	1161 (329)	0.002

Values represent mean (SD) unless otherwise stated. *P* values are result of one-way analysis of variance or χ^2^ test as appropriate comparing the individual clusters. Clusters arranged in progression from non-inverted (cluster 5) to inverted (cluster 4) morphology

There was no association between either PaO_2_ oscillation height (*P* = 0.07) or mean PaO_2_ (*P* = 0.12) and cluster membership.

In an exploratory analysis assessing the effect of intra-animal variability upon cluster membership, SD of the random effects (animal) term was 0.74 clusters. Gradients for fixed effects terms were − 0.06 clusters/kPa (− 0.0077 clusters/mmHg; *P* = 0.03) for PaO_2_/FiO_2_ ratio and 0.065 clusters/% (*P* = 0.002) for pulse pressure variation.

## Discussion

In this pilot study in lung-injured and uninjured pigs undergoing mechanical ventilation, FPCA and *k*-means clustering identified distinct intra-tidal PaO_2_ oscillation morphologies. A progression in the shape of the oscillations was noted, ranging from classically described ventilatory-dependent oscillations [2, 5], which rise in inspiration and decline in expiration (cluster 5), through development of a decline in early expiration (clusters 3, 2 and 1) to fully inverted perfusion-dependent oscillations (cluster 4) [10]. This visual identification of a progression was supported by our analysis of the change in coefficients of the first principal component along the proposed cluster progression (Additional file 6: Fig. S6).

To the best of our knowledge, these PaO_2_ oscillations with two peaks and troughs per breath (clusters 2 and 1) have not been previously described. Therefore, although this was primarily a pilot study to assess the feasibility of the analysis technique, we feel it is justified to hypothesise the aetiology of these oscillations.

The progression of PaO_2_ oscillation morphology was associated with increasing plateau pressure and driving pressure from cluster 5 through 3, 2 and 1 (Table 1). Assuming cluster 4 represents the most extreme manifestation of the development of a reduction in PaO_2_ in early expiration, it could be expected that this trend would continue, but this was not the case. Alternatively, in this cluster the cardiac output was lower than immediately preceding clusters. It may be hypothesised, therefore, that the development of inverted PaO_2_ oscillations is related to relative pulmonary hypoperfusion caused by the interaction of both increased airway pressures and reduced cardiac output. This result is supported by the finding that pulse pressure variation was increased in clusters 1 and 4 (Table 1) as well as a significant association between increasing pulse pressure variation and progression in cluster membership from non-inverted to inverted oscillations in our linear model.

During a sustained rise in intrathoracic pressure, such as is seen with a recruitment manoeuvre, there is a redistribution of pulmonary blood volume from well- to poorly aerated lung regions [24]. Such a redistribution has also been demonstrated during tidal ventilation in pig lung injury models [25]. It is, therefore, possible that the development of inverted oscillations is also related not solely to absolute variation in pulmonary perfusion, but the complex interplay between regional pulmonary aeration and blood flow mediated by variations in mechanical ventilation and cardiac output.

In the intermediate clusters, we demonstrated a rise in PaO_2_ at the start of inspiration, a dip towards the end of inspiration, a further increase in early expiration followed then by a decline towards the end-expiratory value. In some clusters (3 and 2), the expiratory increase was greater than the peak inspiratory PaO_2_ value. At a speculative level, it is possible that a progressively larger proportion of the lung exhibiting zone 2 physiology determined these changes, where positive intrathoracic pressure acts as a Starling resistor on post-capillary venules. Thus, even in the setting of perfect V̇/Q̇ matching, where the pulmonary blood is well-oxygenated during inspiration, only a small amount exits the lung and is seen by the PaO_2_ sensor during inspiration. Upon release of the inspiratory pressure, a large volume of well-oxygenated blood leaves the lung and is seen by the PaO_2_ sensor. With progressive lung injury, however, the effects of intra-tidal variation in V̇/Q̇ matching, as described above, predominate. These hypotheses require confirmation with experiments that directly measure both regional V̇/Q̇ matching and cardiac output throughout the course of a single breath.

The effects of lung injury, as measured by PaO_2_/FiO_2_ ratio upon PaO_2_ oscillation morphology are difficult to ascertain from our data. We did demonstrate a significant association between decreasing PaO_2_/FiO_2_ ratio and a progression in cluster membership from non-inverted to inverted oscillations in our linear model. In addition, there was a statistically significant variation in PaO_2_/FiO_2_ ratio amongst the clusters; however, this variation disappeared in a sensitivity analysis that excluded cluster 4 (inverted oscillations) due it mainly comprising ventilatory conditions from a single animal. In this case the results of such analyses are limited by the small sample size, which was rather chosen to demonstrate the feasibility of the signal analysis methodology and demonstrate novel PaO_2_ oscillation morphologies, rather than understand all potential aetiologies of these morphologies.

A significant strength of the methodology demonstrated here for identification of unique morphologies of PaO_2_ oscillation is that it is not based on any a priori assumptions about the shape of the waveform. FPCA simply generates a series of component functions which, in various combinations, are able to describe the original waveforms [18]. For example, there is no input to the FPCA algorithm to bias it towards generating output waveforms that have peaks and troughs aligned with the switch from the inspiratory to the expiratory phase of mechanical ventilation. The fact that such a switch was associated with a brief dip in the PaO_2_ waveform in clusters 2 and 1 is reassuring in that the algorithm produces results that are physiologically plausible. We chose FPCA over other forms of complex signal analysis, for example, Fourier analysis, because it identifies multiple components of the signal at the same frequency that comprise the PaO_2_ signal. This approach is important, because we hypothesised that the primary input frequency to generate the PaO_2_ oscillations is the respiratory frequency; therefore, the components of multiple harmonics of this frequency (as would be obtained by Fourier analysis) are arguably less relevant than the effect of multiple different signals at the same frequency (e. g. a cyclical R/D signal as distinct from perfusion variation signal). This benefit of FPCA has previously been exploited in analysing electroencephalogram signals to identify multiple components within similar frequency bands [26].

It is possible to simply use the coefficients of the FPCA component signals as clusters. For example, an individual waveform could be composed of 95% of the first principal component and 5% of the second. Therefore, this hypothetical waveform could simply be classified as representing the first principal component. Unfortunately, this is problematic, because the principal component functions are ranked, i. e. the first explains more of the variance in all the signal than the second, which likewise explains more that the third, etc. However, by feeding the coefficients of the principal component functions as inputs to a clustering algorithm [20] it is possible to determine clusters that do not necessarily align with the component functions. For example, it may be possible to identify one cluster which has a 95%/5% split between the first two principal components and a second cluster with a 55%/45% split—both of these are predominantly comprised of the first principal component, but the clustering algorithm is able to differentiate them in a non-biased manner.

Of note, even though we identified 5 unique morphologies of PaO_2_ oscillation in this study, they appear to represent a progression of physiology rather than distinct phenotypes. This is a limitation of the clustering technique, which identifies distinct groups, rather than continuous progressions. The morphologies demonstrated, however, including non-inverted, biphasic, and inverted oscillations should form the basis of further studies aiming to understand the aetiology of each component.

The major limitation with our study is the method used to align the PaO_2_ waveforms with the phases of ventilation. This was based on the assumption that the time delay between a change in ventilation and a change in the PaO_2_ waveform is constant between tidal ventilation and during a prolonged end-expiratory breath hold. Such an assumption may not hold if cardiac output is significantly different between tidal ventilation and during the end-expiratory breath hold. It is plausible that cluster 5 (where PaO_2_ rises in inspiration and declines in expiration) is actually similar to cluster 4 (which declines in inspiration and rises in expiration) shifted along the time axis, and this shift may have been artefactual due to errors in determining the time delay between the airway pressure and PaO_2_ signals. This is less likely to be the case, however, due to the significant differences in physiology seen between the various clusters. If the difference between cluster 5 and cluster 4 is purely related to measuring error, then the physiological variables would be expected to be randomly distributed between the two clusters, rather than distinct as seen in this study.

To reduce the number of animals required for this study, the experiments were performed on animals also involved in a separate study [12], and the choice of lung-injury or not was dependent upon the requirements of this other study. It is unlikely that any significant bias was introduced by this approach, given that the primary grouping variable used in the analyses was PaO_2_ oscillation cluster membership, rather than lung-injury or not. The previous experiment also dictated the maximum sample size available for the current experiments. Overall, we were able to include 73 individual ventilatory conditions in our results, a number which either exceeds or is similar to the that used in previous work on PaO_2_ oscillations, which demonstrated significant inter-group differences [8–10]. Therefore, we think that the number of animals and ventilatory conditions studied was appropriate to separate the effects of common underlying physiologies from any individual animal effect.

A final limitation of this study is that we required PaO_2_ to be greater than 100 mmHg throughout. In ARDS, an average PaO_2_ greater than 105 mmHg is associated with adverse outcomes [27], and therefore, this requirement makes translation of continuous PaO_2_ sensing technology to the bedside less appealing. It should be noted, however, that this requirement was merely to improve the discriminative accuracy of the FPCA and clustering algorithms to detect unique PaO_2_ oscillation morphologies. Once a larger database of potential PaO_2_ morphologies (and their underlying physiologies) is collated, it should be eminently possible to clinically utilise the sensor at lower PaO_2_ values and then compare the collected oscillations (with associated dampening due to haemoglobin desaturation) against the archetypal oscillations presented here when such dampening is absent.

## Conclusions

In this pilot study, we demonstrated the feasibility of FPCA in identifying unique morphologies of PaO_2_ oscillations during mechanical ventilation in a porcine model with different cardiorespiratory physiology. This study was not powered to demonstrate an inter-cluster difference in these physiologies, but aimed to validate the technique and identify PaO_2_ morphologies that warrant further investigation with dynamic measures of lung perfusion (for example, dynamic dual-energy CT [25]). Such investigations would potentially allow separating any cyclical R/D signal in the signal from other signals, including oxygen utilisation during the breath [14] and pulmonary perfusion variations. Such experiments are essential prior to advocating PaO_2_ oscillation analysis as a marker of cyclical R/D, if appropriate.

## Take-home message

Intra-tidal oscillations in arterial oxygen tension have been demonstrated in animal models of the acute respiratory distress syndrome. In this study functional principal component analysis was used to identify unique morphologies of these oscillations which are potentially generated by distinct cardiorespiratory physiologies.

### Supplementary Information

**Additional file 1: Figure S1**. Example of alignment of PaO_2_ signals with the phases of ventilation. The restart of mechanical ventilation following a prolonged breath hold at end-expiration was used to align the PaO_2_ waveform with airway pressure (Paw). At the end of a breath hold, the increase in airway pressure (black arrow) indicated the timepoint when inspiration began. Following this beginning of inspiration, the timepoint at which the PaO_2_ waveform began to rise was visually identified (red arrow). The period between these two timepoints was applied retrospectively to the tidal ventilation prior to the breath hold and used to align the two waveforms. a) The typical case where a discrete change point in the PaO_2_ signal is present. b) The uncommon occurrence where a discrete change point in the PaO_2_ waveform was not obvious. In this case, the intersection between two straight lines representing the gradient of the PaO_2_ decline at the end of the breath hold and the rise during inspiration was used (shown here as dotted red lines).

**Additional file 2: Figure S2**. Trajectories of PaO_2_ during lung lavage for the three lung injured animals. Each line represents a single lavage. Timepoint 0 represents the restart of ventilation at the end of the lavage. Points before this represent the PaO_2_ at the start of the lavage and the PaO_2_ at 30 s intervals after the lavage is provided.

**Additional file 3: Figure S3**. Fraction of variance in the PaO_2_ oscillation waveform data set explained by successive principal components. Bars represent the fraction explained by each individual principal component and the line is the cumulative sum of all principal components up to and including the current. Five principal components were required to explain ≥ 95% of the variance in the PaO_2_ waveforms.

**Additional file 4: Figure S4**. Akaike Information Criterion (AIC) analysis to identify the optimum number of clusters. Given that *k*-means clustering is inherently stochastic, a Monte Carlo simulation was run over 10000 iterations for each individual total number of clusters. Points denote mean and error bars SD of AIC. AIC was optimised with between 3 and 5 clusters.

**Additional file 5:** **Figure S5.** Coefficients of first three principal components grouped by cluster membership (colours) and animal number (shapes).

**Additional file 6: Figure S6**. Effect of progression through clusters from 5 (non-inverted, ventilation-dependent) through cluster 4 (inverted, perfusion-dependent) upon the estimates of each individual functional principal component (PC). In the first principal component a linear progression was noted (*P* < 0.001, *R*^2^ = 0.88). Each individual point represents the PC estimate for a single ventilatory condition.

**Additional file 7: Table S1.** Cluster membership by animal. Values represent number of ventilatory conditions from each animal included in each PaO_2_ cluster.

**Additional file 8: Table S2**. Sensitivity analysis of effect of cluster membership upon cardiorespiratory parameters excluding cluster 4 which was predominantly comprised of ventilatory conditions from a single animal. Values represent mean (SD) unless otherwise stated. P values are result of one-way analysis of variance or χ2 test as appropriate comparing the individual clusters. Clusters arranged as per Table 1 with the exclusion of Cluster 4 (inverted, perfusion-dependent).

## Data Availability

The data sets generated and/or analysed during the current study are not publicly available due to the large size of the complete data sets. They are available from the corresponding author on reasonable request.
