# Supplementary material for: Intra-tidal PaO2 oscillations associated with mechanical ventilation: a pilot study to identify discrete morphologies in a porcine model
Source: Intensive Care Med Exp. 2023 Sep 6;11:60. doi: 10.1186/s40635-023-00544-0 (PMC10482813; doi:10.1186/s40635-023-00544-0)

**Figure S1**. Example of alignment of PaO_2_ signals with the phases of ventilation. The restart of mechanical ventilation following a prolonged breath hold at end-expiration was used to align the PaO_2_ waveform with airway pressure (Paw). At the end of a breath hold, the increase in airway pressure (black arrow) indicated the time point when inspiration began. Following this beginning of inspiration, the time point at which the PaO_2_ waveform began to rise was visually identified (red arrow). The period between these two time points was applied retrospectively to the tidal ventilation prior to the breath hold and used to align the two waveforms. a) The typical case where a discrete change point in the PaO_2_ signal is present. b) The uncommon occurrence where a discrete change point in the PaO_2_ waveform was not obvious. In this case, the intersection between two straight lines representing the gradient of the PaO_2_ decline at the end of the breath hold and the rise during inspiration was used (shown here as dotted red lines).


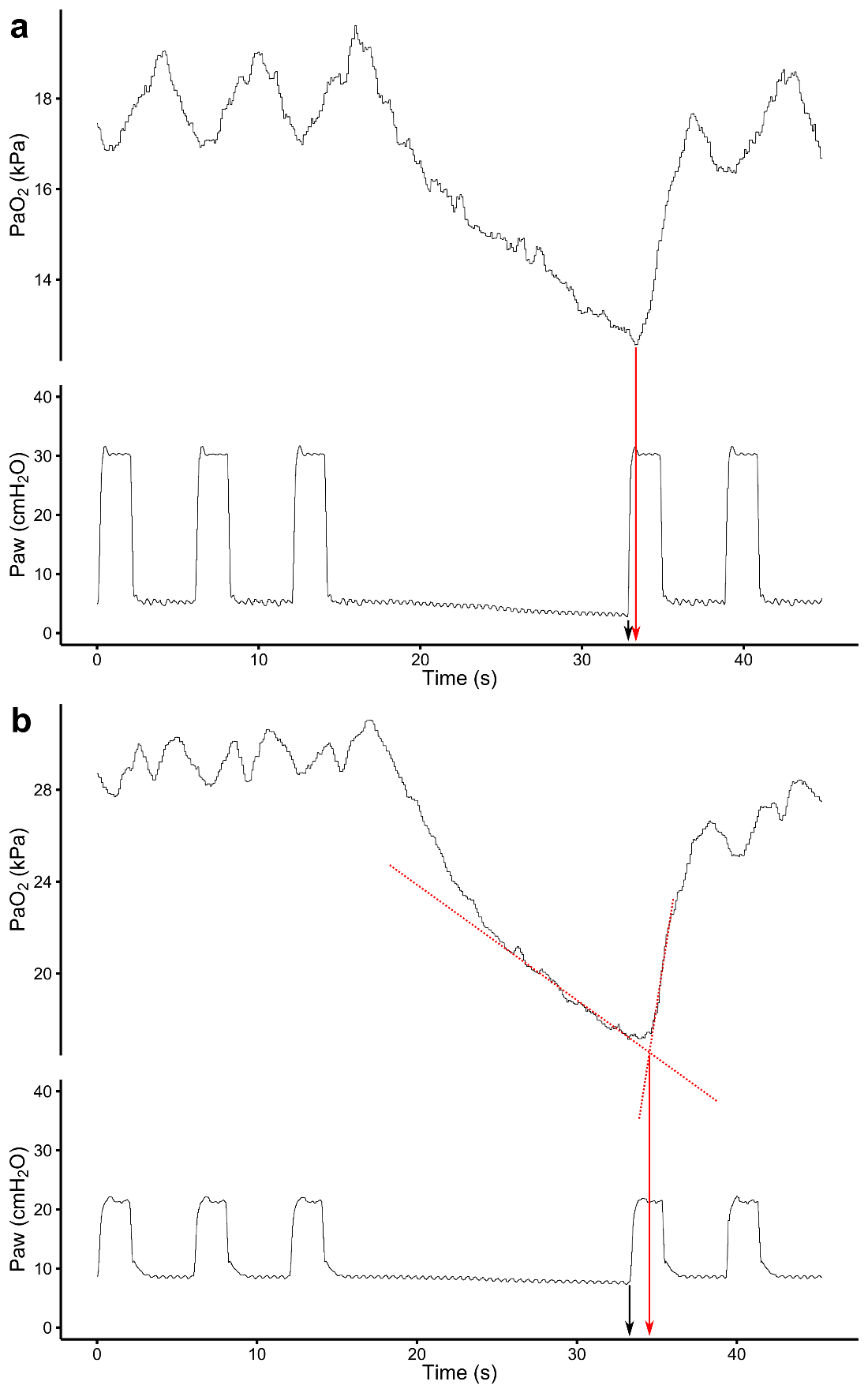

Supplement: Supplementary file 1 — Additional file 1: Figure S1. Example of alignment of PaO2 signals with the phases of ventilation. The restart of mechanical ventilation following a prolonged breath hold at end-expiration was used to align the PaO2 waveform with airway pressure (Paw). At the end of a breath hold, the increase in airway pressure (black arrow) indicated the timepoint when inspiration began. Following this beginning of inspiration, the timepoint at which the PaO2 waveform began to rise was visually identified (red arrow). The period between these two timepoints was applied retrospectively to the tidal ventilation prior to the breath hold and used to align the two waveforms. a) The typical case where a discrete change point in the PaO2 signal is present. b) The uncommon occurrence where a discrete change point in the PaO2 waveform was not obvious. In this case, the intersection between two straight lines representing the gradient of the PaO2 decline at the end of the breath hold and the rise during inspiration was used (shown here as dotted red lines). [file 40635_2023_544_MOESM1_ESM.docx]
