# Supplementary material for: Intra-tidal PaO2 oscillations associated with mechanical ventilation: a pilot study to identify discrete morphologies in a porcine model
Source: Intensive Care Med Exp. 2023 Sep 6;11:60. doi: 10.1186/s40635-023-00544-0 (PMC10482813; doi:10.1186/s40635-023-00544-0)

**Figure S2**. Trajectories of PaO_2_ during lung lavage for the three lung injured animals. Each line represents a single lavage. Time point 0 represents the restart of ventilation at the end of the lavage. Points before this represent the PaO_2_ at the start of the lavage and the PaO_2_ at 30 s intervals after the lavage is provided.


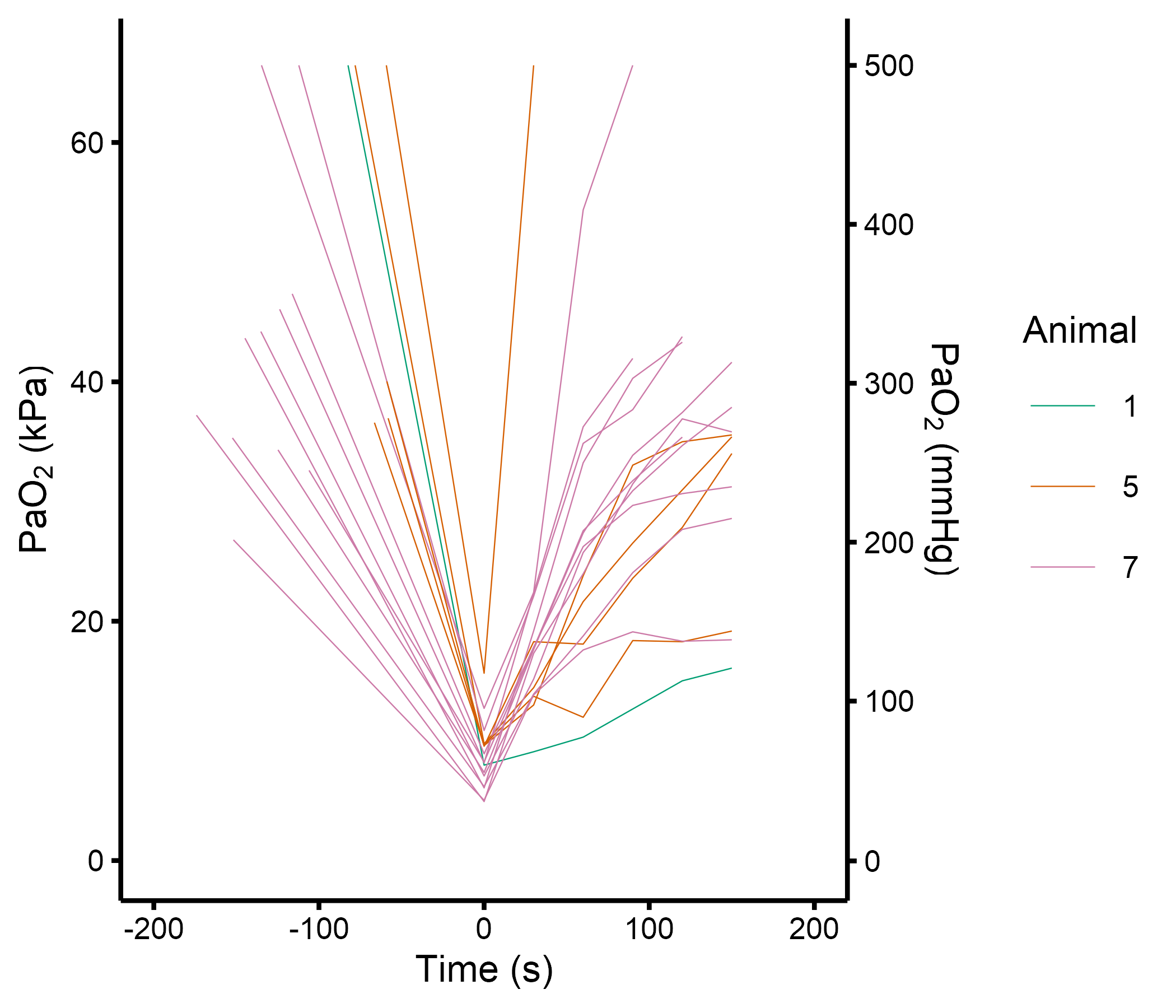

Supplement: Supplementary file 2 — Additional file 2: Figure S2. Trajectories of PaO2 during lung lavage for the three lung injured animals. Each line represents a single lavage. Timepoint 0 represents the restart of ventilation at the end of the lavage. Points before this represent the PaO2 at the start of the lavage and the PaO2 at 30 s intervals after the lavage is provided. [file 40635_2023_544_MOESM2_ESM.docx]
