# Supplementary material for: Intra-tidal PaO2 oscillations associated with mechanical ventilation: a pilot study to identify discrete morphologies in a porcine model
Source: Intensive Care Med Exp. 2023 Sep 6;11:60. doi: 10.1186/s40635-023-00544-0 (PMC10482813; doi:10.1186/s40635-023-00544-0)

**Figure S3**. Fraction of variance in the PaO_2_ oscillation waveform dataset explained by successive principal components. Bars represent the fraction explained by each individual principal component and the line is the cumulative sum of all principal components up to and including the current. Five principal components were required to explain ≥95% of the variance in the PaO_2_ waveforms.


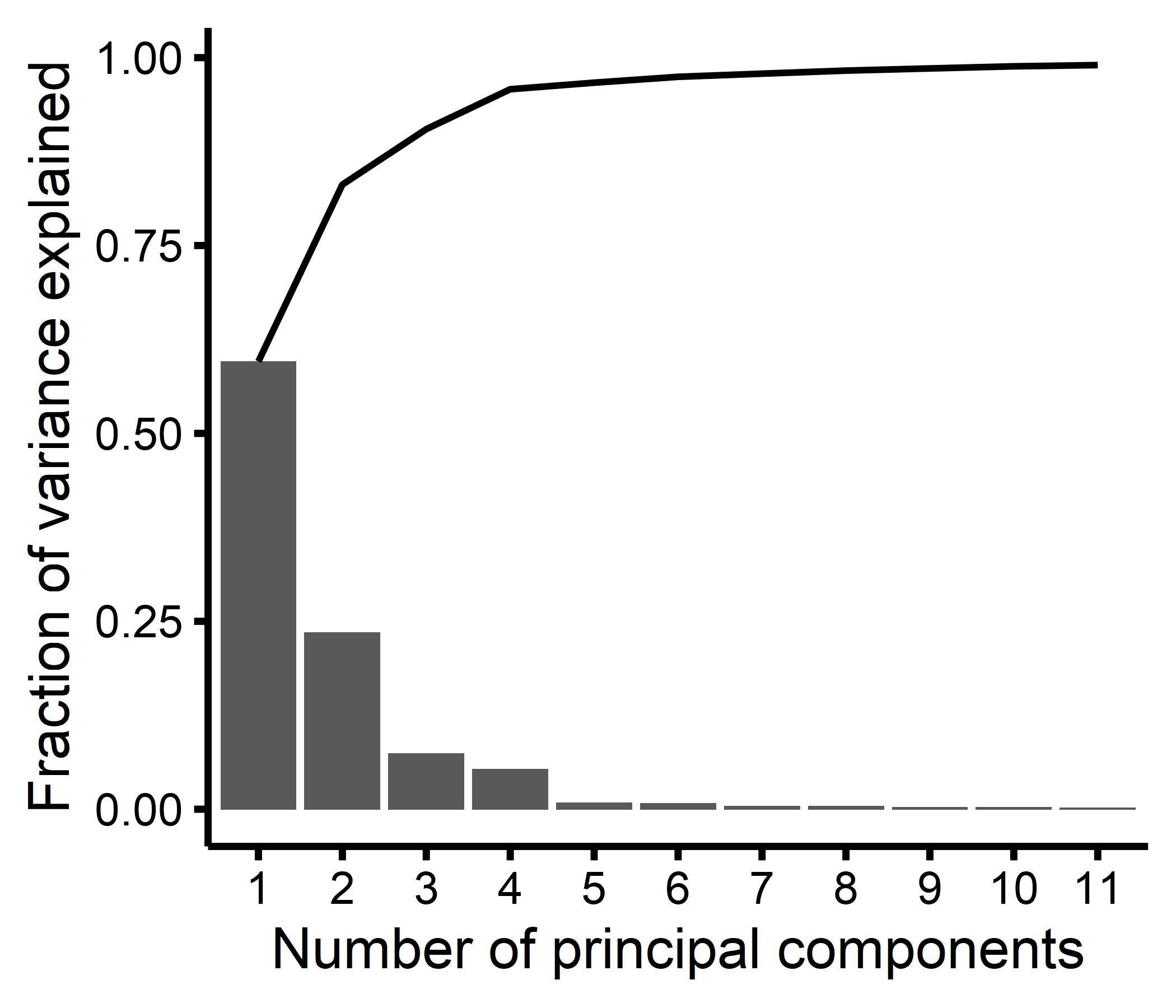

Supplement: Supplementary file 3 — Additional file 3: Figure S3. Fraction of variance in the PaO2 oscillation waveform data set explained by successive principal components. Bars represent the fraction explained by each individual principal component and the line is the cumulative sum of all principal components up to and including the current. Five principal components were required to explain ≥ 95% of the variance in the PaO2 waveforms. [file 40635_2023_544_MOESM3_ESM.docx]
