# Supplementary material for: Intra-tidal PaO2 oscillations associated with mechanical ventilation: a pilot study to identify discrete morphologies in a porcine model
Source: Intensive Care Med Exp. 2023 Sep 6;11:60. doi: 10.1186/s40635-023-00544-0 (PMC10482813; doi:10.1186/s40635-023-00544-0)

**Figure S4**. Akaike Information Criterion (AIC) analysis to identify the optimum number of clusters. Given that k-means clustering is inherently stochastic, a Monte Carlo simulation was run over 10000 iterations for each individual total number of clusters. Points denote mean and error bars SD of AIC. AIC was optimised with between 3 and 5 clusters.


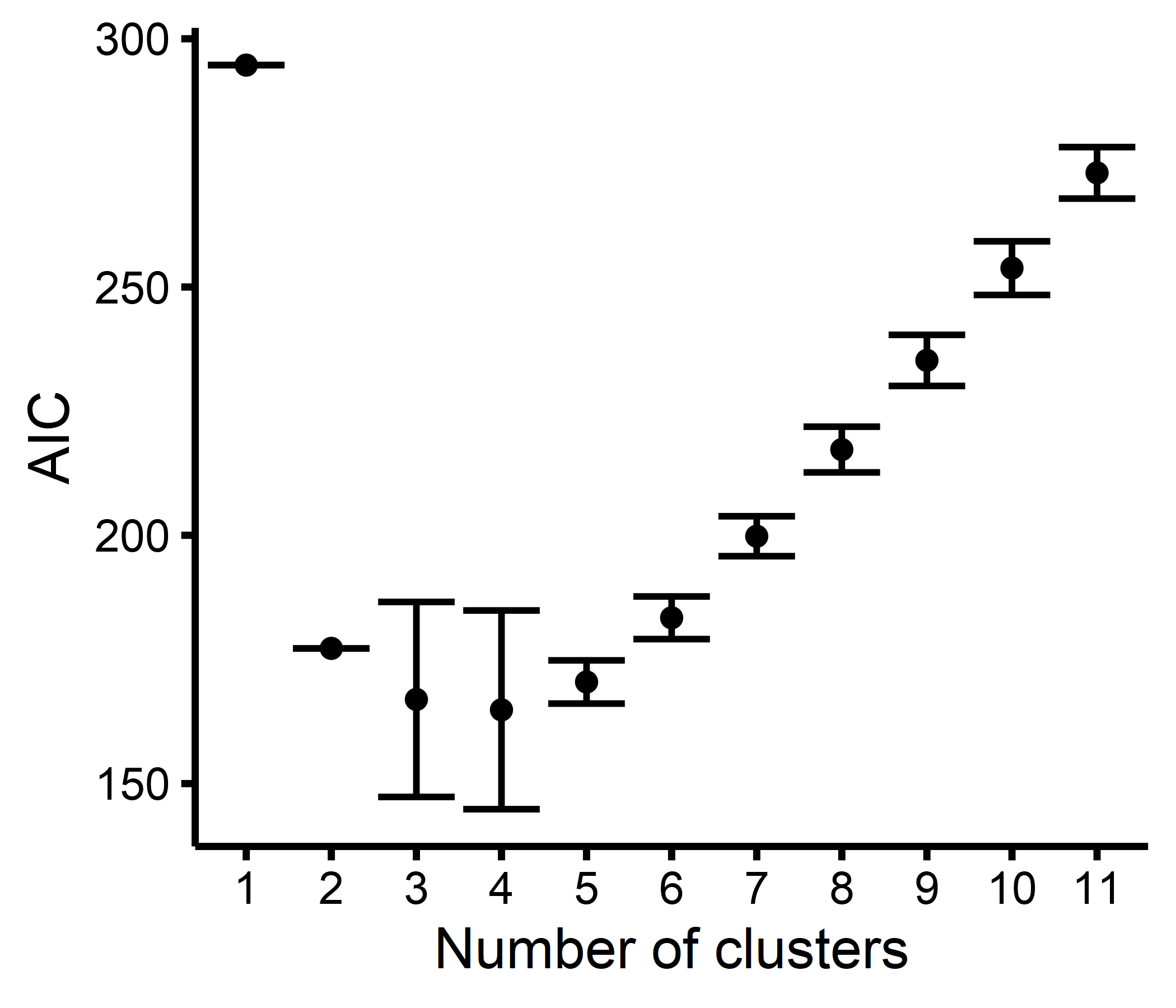

Supplement: Supplementary file 4 — Additional file 4: Figure S4. Akaike Information Criterion (AIC) analysis to identify the optimum number of clusters. Given that k-means clustering is inherently stochastic, a Monte Carlo simulation was run over 10000 iterations for each individual total number of clusters. Points denote mean and error bars SD of AIC. AIC was optimised with between 3 and 5 clusters. [file 40635_2023_544_MOESM4_ESM.docx]
