# Supplementary material for: Intra-tidal PaO2 oscillations associated with mechanical ventilation: a pilot study to identify discrete morphologies in a porcine model
Source: Intensive Care Med Exp. 2023 Sep 6;11:60. doi: 10.1186/s40635-023-00544-0 (PMC10482813; doi:10.1186/s40635-023-00544-0)
Supplement: Supplementary file 5 — Additional file 5: Figure S5. Coefficients of first three principal components grouped by cluster membership (colours) and animal number (shapes). [file 40635_2023_544_MOESM5_ESM.html]

Fig S5. Coefficients of first three principal components grouped by cluster membership (colours) and animal number (shapes).
