# Supplementary material for: Intra-tidal PaO2 oscillations associated with mechanical ventilation: a pilot study to identify discrete morphologies in a porcine model
Source: Intensive Care Med Exp. 2023 Sep 6;11:60. doi: 10.1186/s40635-023-00544-0 (PMC10482813; doi:10.1186/s40635-023-00544-0)

**Figure S6**. Effect of progression through clusters from 5 (non-inverted, ventilation-dependent) through cluster 4 (inverted, perfusion-dependent) upon the estimates of each individual functional principal component (PC). In the first principal component a linear progression was noted (*P*<0.001, *R*^2^=0.88). Each individual point represent the PC estimate for a single ventilatory condition.


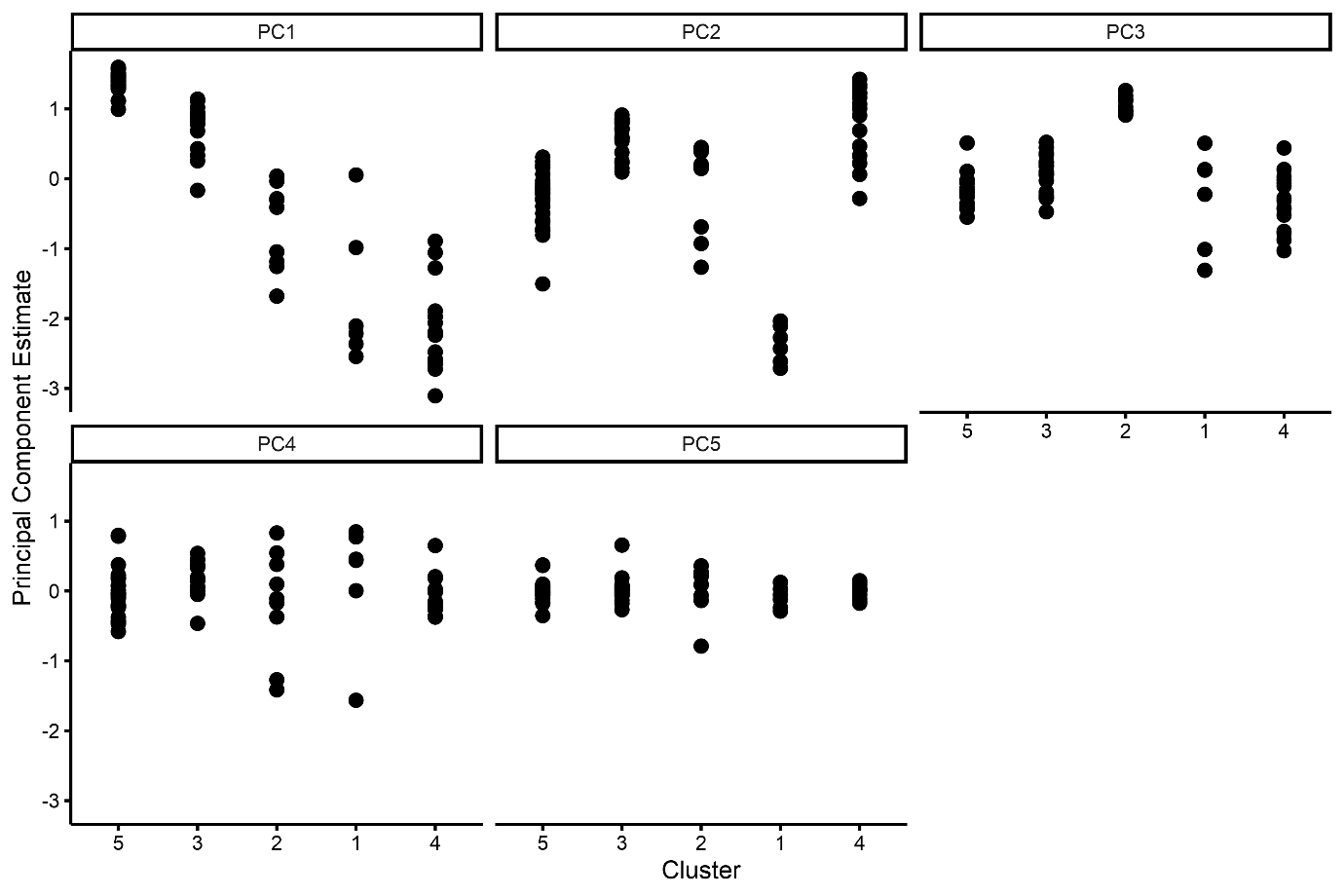

Supplement: Supplementary file 6 — Additional file 6: Figure S6. Effect of progression through clusters from 5 (non-inverted, ventilation-dependent) through cluster 4 (inverted, perfusion-dependent) upon the estimates of each individual functional principal component (PC). In the first principal component a linear progression was noted (P < 0.001, R2 = 0.88). Each individual point represents the PC estimate for a single ventilatory condition. [file 40635_2023_544_MOESM6_ESM.docx]
