# Supplementary material for: Intra-tidal PaO2 oscillations associated with mechanical ventilation: a pilot study to identify discrete morphologies in a porcine model
Source: Intensive Care Med Exp. 2023 Sep 6;11:60. doi: 10.1186/s40635-023-00544-0 (PMC10482813; doi:10.1186/s40635-023-00544-0)
Supplement: Supplementary file 7 — Additional file 7: Table S1. Cluster membership by animal. Values represent number of ventilatory conditions from each animal included in each PaO2 cluster. [file 40635_2023_544_MOESM7_ESM.docx]

**Table S1**

Cluster membership by animal. Values represent number of ventilatory conditions from each animal included in each PaO_2_ cluster.

| **PaO_2_ Cluster** | **Uninjured Animals** | | | | **Lung-injury Animals** | | |
| --- | --- | --- | --- | --- | --- | --- | --- |
|  | **Animal 2** | **Animal 3** | **Animal 4** | **Animal 6** | **Animal 1** | **Animal 5** | **Animal 7** |
| 1 | 0 | 1 | 0 | 2 | 2 | 1 | 0 |
| 2 | 0 | 1 | 0 | 3 | 3 | 2 | 0 |
| 3 | 6 | 1 | 4 | 2 | 2 | 3 | 0 |
| 4 | 2 | 0 | 1 | 0 | 0 | 0 | 12 |
| 5 | 3 | 2 | 7 | 5 | 5 | 3 | 0 |
