# Supplementary material for: Intra-tidal PaO2 oscillations associated with mechanical ventilation: a pilot study to identify discrete morphologies in a porcine model
Source: Intensive Care Med Exp. 2023 Sep 6;11:60. doi: 10.1186/s40635-023-00544-0 (PMC10482813; doi:10.1186/s40635-023-00544-0)
Supplement: Supplementary file 8 — Additional file 8: Table S2. Sensitivity analysis of effect of cluster membership upon cardiorespiratory parameters excluding cluster 4 which was predominantly comprised of ventilatory conditions from a single animal. Values represent mean (SD) unless otherwise stated. P values are result of one-way analysis of variance or χ2 test as appropriate comparing the individual clusters. Clusters arranged as per Table 1 with the exclusion of Cluster 4 (inverted, perfusion-dependent). [file 40635_2023_544_MOESM8_ESM.docx]

**Table S2**

|  | **Cluster 5** | **Cluster 3** | **Cluster 2** | **Cluster 1** | **P** |
| --- | --- | --- | --- | --- | --- |
|  | *Non-inverted*  *Ventilation-dependent* | | *Intermediate* | |  |
| Lung injured/  uninjured ratio (% injured) | 8/17 (32%) | 5/13 (28%) | 5/4 (56%) | 3/3 (50%) | 0.44 |
| PaO_2_/FiO_2_ Ratio (kPa) | 44 (11) | 43 (10) | 39 (9) | 39 (9) | 0.47 |
| PaO_2_/FiO_2_ Ratio (mmHg) | 332 (84) | 323 (74) | 291 (66) | 294 (70) | 0.47 |
|  |  |  |  |  |  |
| Tidal Volume (mL) | 270 (114) | 333 (88) | 396 (117) | 397 (134) | 0.009 |
| PEEP (cmH_2_O) | 9 (3) | 8 (2) | 9 (3) | 11 (1) | 0.04 |
| Plateau pressure (cmH_2_O) | 19 (5) | 20 (5) | 26 (6) | 30 (3) | <0.001 |
| Driving pressure (cmH_2_O) | 10 (4) | 12 (5) | 17 (6) | 18 (3) | <0.001 |
| Dynamic Compliance (mL/cmH_2_O) | 28 (8) | 28 (8) | 24 (9) | 22 (8) | 0.49 |
| Mechanical Power (J/min) | 5.3 (3.8) | 6.5 (2.6) | 9.5 (3.3) | 12.4 (3.1) | <0.001 |
|  |  |  |  |  |  |
| Cardiac Output (L/min) | 4.74 (1.13) | 4.20 (1.21) | 5.36 (0.93) | 5.39 (0.95) | 0.04 |
| Heart Rate (/min) | 125 (27) | 110 (17) | 146 (26) | 125 (7) | 0.007 |
| Mean Arterial Pressure (mmHg) | 102 (19) | 98 (18) | 100 (12) | 92 (25) | 0.73 |
| Pulse Pressure Variation (%) | 12.3 (5.7) | 12.6 (5.3) | 12.7 (4.0) | 20.8 (4.2) | 0.02 |
| Central Venous Pressure (mmHg) | 15 (2) | 14 (2) | 14 (1) | 13 (2) | 0.13 |
| Pulmonary Artery Pressure (mmHg) | 27 (5) | 26 (5) | 25 (4) | 24 (5) | 0.74 |
| Systemic Vascular Resistance (dynes.s/cm^5^) | 1546 (399) | 1724 (430) | 1348 (356) | 1246 (556) | 0.07 |

Sensitivity analysis of effect of cluster membership upon cardiorespiratory parameters excluding cluster 4 which was predominantly comprised of ventilatory conditions from a single animal. Values represent mean (SD) unless otherwise stated. P values are result of one-way analysis of variance or χ^2^ test as appropriate comparing the individual clusters. Clusters arranged as per Table 1 with the exclusion of Cluster 4 (inverted, perfusion-dependent).
